# Anti-social behavior and soccer identities: different continents, same mindset?

**DOI:** 10.1080/15298868.2024.2423829

**Published:** 2024-11-12

**Authors:** Martha Newson, Linus Peitz, Susilo Wibisono, Jorge Knijnik, Fiona White, Harvey Whitehouse

**Affiliations:** a Institute of Lifecourse Development, University of Greenwich, London, UK; b Centre for the Study of Social Cohesion, University of Oxford, Oxford, UK; c SSPSSR, University of Kent, Canterbury, UK; d Psychology, University of Queensland, Brisbane, Australia; e Institute for Culture and Society, Western Sydney University, Penrith, Australia; f School of Psychology, University of Sydney, Penrith, Australia

**Keywords:** Identity fusion, ultras, fandom, social dominance orientation, intergroup psychology

## Abstract

Although most soccer fans support their teams peacefully, anti-social fan behavior continues to appear across the globe. We tested the roles of identity fusion and membership to an extreme fan group (ultras) in explaining fan disorder in two understudied contexts: Indonesia (Study 1) and Australia (Study 2). Incidents of violence and antisocial behavior were rarely reported among general Indonesian (9%) or Australian fans (6%) but were significantly higher among their respective ultras groups (37%; 20%). Identity fusion predicted antisocial behavior, especially when combined with fan group membership. Fusion explained anti-social behaviors better than identification or social dominance orientation. Understanding the motivators of intergroup violence is crucial to devise more effective ways of channeling cohesion among group members into peaceful forms of prosocial action.

Soccer has a long history of inter-group violence and the problem of how to deal with it has sparked public and political debate since at least the 1960s (Doidge, 2013; Giulianotti, 1994). Soccer teams compete on the pitch, but fans also see themselves as engaged in that competition vicariously: stadia and surrounding areas become fans’ own territories; soccer rivalries with other clubs last for generations; and club success or defeat can be transformative at a personal level (Karanfil, 2017; Newson et al., 2016). In soccer, violence toward rivals (outgroup violence) even risks injury or death to the perpetrators themselves (Scalia, 2009; Testa & Armstrong, 2010).

Across many types of conflict, including militia and gang-led conflicts, group structures include more extreme, tightly bonded subgroups that are willing to fight for – and defend – the group with substantial risks to personal safety. Individuals who spend time in subgroups are likely to experience intense, perhaps traumatic events, events that feel uniquely shaping for their group. This might include prejudice and attacks, victories over rival subgroups, and the rise and fall of pivotal figures within the subgroup who are relatively unknown outside of the group.

These sub-groups also exist within the world of soccer. Cultural variation between soccer sub-groups varies globally, though some fan cultures are also transnational (Doidge et al., 2019). For instance, traditionally, there have been soccer hooligans who form *firms* in the UK, *torcidas oragnizadas* in Brazil, *barras bravas* in Argentina, and the rest of Latin America, and *ultras* in Italy and Southern Europe (Spaaij, 2007). The ultras movement is now one of the largest fan movements globally, with groups present in countries as different as Indonesia (Fuller & Junaedi, 2018) and Australia (Knijnik & Newson, 2021). Despite the spread of this movement, these regions have been relatively overlooked in soccer literature, which this research aims to address.

Ultras are here defined as hardcore fan subgroups. Though culturally different from hooligan subcultures in Northern Europe, both ultras and hooligans refer to socially distinctive fan subcultures that engage in regular and collective competitively aggressive behaviors, primarily with rival peers (Spaaij, 2007). As with hooligans, the aggressive, and sometimes violent, behaviors observed among ultras tend to be organized (to varying degrees) rather than spontaneous as would befit general fans to the extent that ultras have been described as militant by some (Spaaij, 2007), a description that cannot be applied to general fans.

As with many subgroups, the distinction between general fans and ultras as more extreme fans reflects an ardent, internally homogeneous subgroup within a more heterogeneous, flexible general population. Ultras are known for their intense dedication to the club and their fellow fans, often pledging large amounts of time and effort toward elaborate displays of commitment and group identity, deeply linked to masculine and national identities, including highly co-ordinated visual displays (e.g., using pyrotechnics) and vociferous chanting and singing (Grodecki, 2022; Winskowski, 2022). Simultaneously, ultras are associated with violence and anti-social behavior, oft-expressed in intergroup rivalries with fans of other teams (Doidge & Lieser, 2018; Doidge et al., 2020), but sometimes even toward their own team (Mozartsport, 2022; Uersfeld, 2021).

Importantly, while the ultras movement may be transnational, fan groups vary substantially; each exhibiting unique characteristics relating to their socio-political composition, historic background, or contemporary appearance (Spaaij, 2007). As such, soccer violence cannot be reduced to the presence of ultras groups and instead depends on various interrelated individual, social, and situational factors (for examples, see Giulianotti, 1994; Spaaij, 2014; van Hiel et al., 2007). Nonetheless, intergroup conflict – with or without the presence of physical violence – is still a hallmark feature of much of the ultras movement, as is made clear by many ultras groups’ social media content and the use of banning orders by local authorities (Hallinan & Hughson, 2008; Knijnik, 2018a, 2018b, 2020).

To try and capture something of the breadth of ultras culture, we worked with Indonesian and Australian fans. Indonesia, as the largest Muslim democracy in the world, has cultural resonance with parts of South-East Asia, the Middle East, and North Africa, where Islam-centered masculinities dominate (Azra, 2008), parts of the soccer world that are under-researched. By way of comparison, Australia encapsulates many of the cultural norms associated with soccer masculinities in traditionally Christian-centric European cultures, largely comprising people of recent North Western, Eastern, or Southern European descent. Using these two regions thus represents two of the most dominant global meaning systems, embedded within which are cultural understandings of masculinity and group cohesion. Sport, particularly soccer, is the ideal context to understand this spectrum of cultural variation (Amara, 2008), which will help international sports and safety organizations to better strategize interventions and security measures.

## Mechanisms of anti-social behaviour in soccer

Research into the underlying mechanisms of soccer violence and hostility has identified many contributing factors, including fans’ physiology (e.g., cortisol levels, van Der Meij et al., 2015), personality (van Hiel et al., 2007), substance abuse (Ayres & Treadwell, 2012; Newson, 2021), media coverage (Garland & Rowe, 1999), referee performance, and even the weather (Heydarinejad & Gholami, 2012). However, the most common approach has emphasized the role of groups and specifically sub-groups, by exploring cross-national similarities in the construction of fan identities (Hodges, 2016; Spaaij, 2007).

Most notably, this approach has explained fan violence in the context of the Elaborated Social Identity Model (ESIM) of crowd behavior (Stott et al., 2008, 2020), which has influenced policing strategies in Sweden (Stott et al., 2016, 2019). The ESIM proposes that in anonymous group settings (e.g., mass sport events), depersonalization increases the salience of social categories and group membership, and individuals are more likely to adopt behaviors in line with a salient group identity (Reicher, 2001).

Applying this framework to soccer violence, research has shown how the perception of violence as a legitimate group behavior among fan groups can change in response to police tactics, i.e., being treated as “hooligans” increases fans’ likelihood to adopt such a group identity and engage in group normative behaviors, i.e., violence (see Stott & Pearson, 2007; Stott et al., 2001, 2008). This line of work has demonstrated considerable strength in explaining the interplay between group memberships interplay and situational factors. Here, we seek to add to this model both theoretically and empirically by explaining *individual* differences in fan violence, and why some fans and subgroups are more violent than others, providing nuance to the ESIM from new (and under-represented) populations.

## Identity fusion theory and extreme group behaviours

One established individual predictor of extreme group behavior across many diverse populations is “identity fusion” (Swann & Buhrmester, 2015; Swann et al., 2012). This particularly powerful form of group bonding describes a visceral “oneness” felt with one’s group (Swann et al., 2009, 2012). This is conceptually distinct from in-group identification, which is a more anonymous form of social cohesion that is context-dependent and “switched off” when one’s individual sense of self is active (Tajfel & Turner, 1979). As such, fusion is more potent, as the group’s strength and one’s personal agency become immersed in one another (Bortolini et al., 2018; Swann et al., 2012; White et al., 2021).

Many of us are likely to be “fused” with our families (Swann et al., 2014), the people we likely share our biology and much of our most important experiences with (Whitehouse, 2018). Similarly, some fans will fuse to the “soccer family.” Just as an individual may be willing to lay down their lives to save a close family member, a fused fan is willing to lay down their lives to save other fans in peril when they perceive those other fans to be like their own brothers and sisters (Newson, 2019; Newson et al., 2021).

Research has demonstrated that “fused” fans report an increased willingness to fight and die for other fans in both Brazil and Poland (Bortolini et al., 2018; Kossakowski & Besta, 2018), as well as report more incidents of fan-disorder in Australia (White et al., 2021). In Brazil, fused fans belonging to extreme subgroups (*torcidas organizadas)* were particularly likely to report a violent history against rival fans and to the police, compared to weakly fused or general fans (Newson et al., 2018). Importantly, this violence was targeted toward specific outgroups who could potentially cause a threat to the fan group’s physical safety or reputation.

Fusion theory can thus add another layer to the understanding of intergroup conflict among ultras as a deep dedication to the group, a dedication that also has the potential for positive pro-group outcomes. This approach complements the ESIM, offering an explanation at an individual level, as traditional social identity theory perspectives rarely include relational processes in crowd dynamics (Swann et al., 2009).

## Fusion and alternative predictors of extreme group behaviours

Examining the link between fusion and anti-social behavior raises questions regarding alternative predictors of intergroup violence and conflict. Two notable candidates here are group identification (Brewer, 2001; Postmes et al., 2013) and Social Dominance Orientation (SDO) (Levin et al., 2003; Pratto et al., 1994). Ingroup identification has already been well investigated in relation to identity fusion (see introduction, Bortolini et al., 2018; Swann et al., 2009; White et al., 2021; Whitehouse & Lanman, 2014). Here, we seek to support previous literature by examining whether fusion outperforms identification to predict anti-social behavior in soccer.

Furthermore, intergroup relations are negatively impacted by people with high levels of SDO, i.e., a preference for high-status groups to dominate lower-status groups, including more levels of competition and prejudice (Duckitt & Sibley, 2010; Maunder et al., 2020; Ho et al., 2015), making it an ideal candidate to explain extreme intergroup behaviors associated with identity fusion. Research examining fusion, SDO, and extremism has shown fusion to outperform SDO as a predictor of political violence (Besta et al., 2015; Kunst et al., 2019), but to our knowledge, this is the first study to investigate these factors in the context of sports fan anti-social behavior.

## The present research

Here, we examine anti-social behavior among soccer fans and investigate potential differences between ultras and general fans. Specifically, we hypothesize that ultras, compared to general fans, will report more engagement in extreme behaviors (H1), i.e., higher willingness to self-sacrifice (Study 1), past violent behavior (Study 1), and anti-social behavior (Study 2). Furthermore, we explore the role of group alignment in connecting subgroup membership and anti-social behavior. We hypothesize that levels of extreme group behaviors will be more prevalent among soccer fans who are fused to their team (H2a). Furthermore, we predict that the effect of fan type (ultras vs general) on extreme group behaviors will be dependent on (i.e., moderated by) fusion (Study 1 & 2; H2b). Lastly, we hypothesize that fusion will be a better predictor of anti-social behavior (Study 2) than related predictors of intergroup conflict (i.e., ingroup identification and SDO).

We test these hypotheses across two studies, with distinct populations of ultras, one with an old and violent history of soccer fandom (Indonesia; Study 1, *n* = 86) and the other with a more recent, peaceful history (Australia; Study 2 *n* = 202). The sample size of 86 Indonesian football fans, with half identified as violent supporters, is justified despite being relatively small due to the challenges of accessing this under-represented group in field research. While a larger sample would enhance generalizability and statistical power, the exploratory nature of the study and the ecological validity of real-world data collection offer valuable insights. The inclusion of a second, well-powered study with Australian fans helps offset limitations in the Indonesian sample, though the findings should be interpreted with caution due to potential issues with statistical power, precision, and cultural generalizability.

## Transparency and openness

We report how we determined our sample size, all data exclusions (if any) and all measures in the study. All data are available on OSF. Data were analyzed using the IBM SPSS software package, version 29. This study’s design and its analysis were not pre-registered.

## Method: study 1

### Sample

We recruited 100 Indonesian soccer fans to complete a pen and paper survey about their fan identities. The sample size was determined as a feasible goal within the constraints of fieldwork in this area and within the available research period. Post hoc power analyses in G*power at the standard .05 alpha error probability (Faul et al., 2009) indicated that all analyses reached power (>.90). Indonesian collaborators, with specialist knowledge and contacts gathered these data in the field, e.g., at meeting points and participants’ homes. This method allowed access to a diverse range of participants who may not have come into a laboratory. All participants supported a popular Indonesian team, Persib Bandung. Half the participants self-identified as ultras due to their membership of long-established ultras fan groups, particularly “The Vikings,” associated with soccer violence.

Participants were incentivized with a souvenir (i.e., stationary) for taking part in the research. Fourteen fans were found to be under 18 and were removed from the dataset. The final sample consisted of 86 fans, including general fans (*n* = 45) and ultras (*n* = 41). Men were over-represented in both samples (>86%), reflecting the fact that men in Indonesia are more commonly involved with soccer than women (Kusuma & Octastefani, 2022) – as tends to be the case globally due to cultural norms, the historical male dominance in the sport, and the perception of football as a male-centered activity (Pfister & Pope, 2018).

The sample was relatively young (*M*_age_ = 25.09, *SD* = 5.82, range = 18–47) and most participants ranked themselves with a middling income (91%). One soccer fan reported being Hindu, whereas all other participants identified as Muslim. Ethical approval was obtained from the [REDACTED] for ethical approval of all research involving human participants (B-361/Un.2/L.03/PN.00/8/2017).

### Measures

Due to time constraints and the sensitive nature of the fieldwork, brief measures were used wherever possible (e.g., a pictural single-item measure of fusion instead of a multi-item verbal scale). First, we asked participants demographic data and group affiliation. To test group alignment, we used a version of the pictorial identity fusion scale (Swann et al., 2009), with reference to participants’ soccer clubs. Here, participants selected the image best representing their relationship to their group from a series of increasingly overlapping circles, with option F showing a small circle (Self) completely immersed in a larger circle (Group). As data appeared to be bimodally distributed in line with previous research (Swann et al., 2009), participants who selected option F were considered “fused” (1), others were not fused (0). To assess personally costly pro-group behaviors, a reduced three-item version of fight and die measure was used (“*I would fight someone physically threatening a fan of my team;” “I’d do anything to protect fans of my team”*; and *“I would sacrifice my life if it saved another fan of my team’s”)* (Swann et al., 2009), on a Likert-type scale of 1–7 (strongly disagree vs. strongly agree), *α* = .77. We asked whether participants had ever physically attacked or injured someone for being a fan of the opposition (yes = 1/no = 0). All measures were translated and back-translated by bilingual Indonesian-English speakers with post-graduate English qualifications. A native English speaker checked back-translations for accuracy, and any discrepancies were translated and back-translated again until agreement was met.

## Results: study 1

One-third of fans were fused to their soccer team, and less than a quarter reported having engaged in fan violence in the past (see Table S1 in the SI for descriptives and correlations). Fusion correlated significantly with fans’ willingness to self-sacrifice for their group, and with being an ultra. Being an ultra also correlated with extreme behaviors (past violence) and willingness to self-sacrifice.

### Predicting engagement in violent behaviour

We investigated whether general fans (*n* = 45) or ultras (*n* = 41) had previously, or were willing to, engage in violence against rivals. Ultras reported significantly more fusion, willingness to fight and die and past violence than general fans (Table 1).

**Table 1.** Comparing key variables between general fans and ultras in study 1.

Variables	General fans *M* (*SD*)/ %/N	Ultras *M* (*SD*)/ %/*N*	Test
Fusion	13%	59%	χ^2^ (86, 1) = 19.30, *p* <.001, φ_*c*_ = .47
Fight and die	3.93 (1.34)	5.32 (1.22)	*t*(84) = −4.99, *p* < .001, *d* = −1.08
Past violence	9%	37%	χ^2^ (86, 1) = 9.56, *p* = .002, φ_*c*_ = .33

Note. For continuous measures we present means (*M*) and standard deviations (*SD*), for binary measures, we present shares (%) of the sample populations *n* coded as 1 out of the total population *N*.

**Figure 1.**
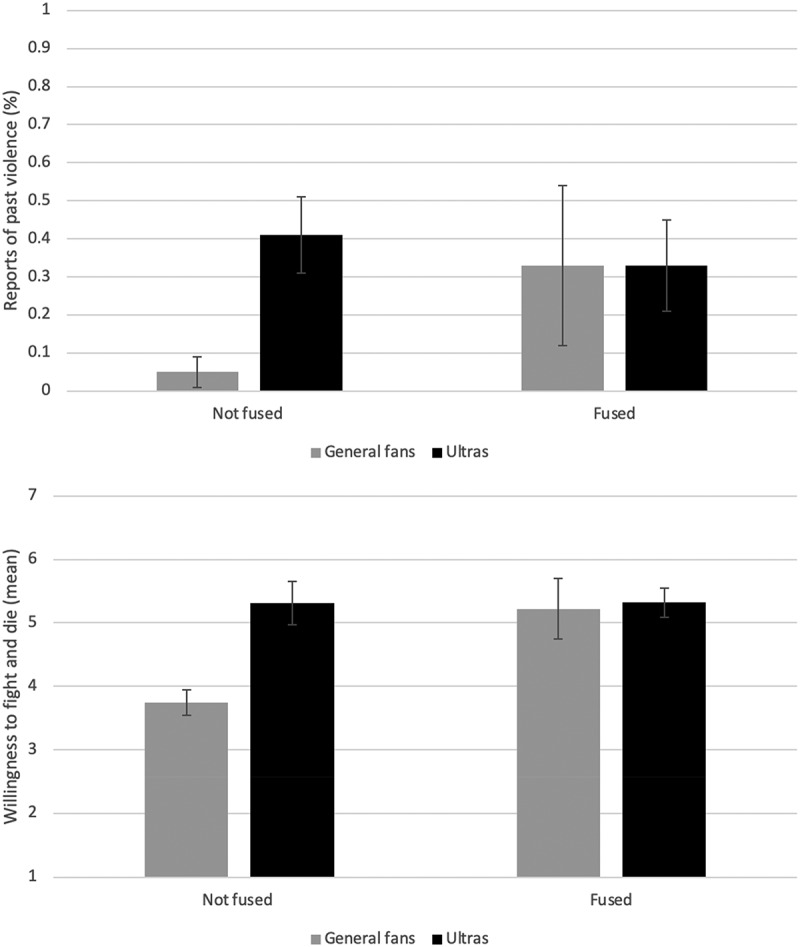
Indonesian Fans’ self-reported past violence and future willingness to fight and die.

*Note*. Self-reported past violence was measured as a binary variable (yes/no), such that the bars represent the share of participants across groups who reported having previously engaged in violence (0–1). Willingness to fight and die was measured on a Likert scale (1 = *strongly disagree*, 7 = *strongly agree*). Error bars show *standard errors.*

Using logistic regression analysis, we examined predictors of violent behavior simultaneously and also tested for interactions between fusion and type of fan (general vs ultras) to predict past violence (physically attacking someone for being a fan of the opposition). Fusion (OR = 15.16, 95% CIs [1.323, 173.770], *p* = .029) and fan type (OR = 16.36, 95% CIs [2.451, 109.232], *p* = .004) both predicted past violence, but this was qualified by their 2-way interaction (OR = 0.04, 95% CIs [.002, .678], *p* = .026), *X*^2^(7) = 23.77, Nagelkerke *R*^2^ = .37, *p <* .001 (see Table S2, SI for full results). Specifically, general fans were significantly more likely to have engaged in past violence when they were fused compared to when they were not fused, whereas ultras reported violence whether they were fused or not (Figure 1). There was a main effect of age (OR = 1.12, 95% CIs [1.016, 1.240], *p* = .023), as older fans were more likely to report past violence, but no effect of gender (OR = 0.00, *p* = .999).

Next, we ran a linear regression model and examined willingness to fight and die for the group based on the same predictors. The trend was similar with main effects of fusion (B = 1.46, SE = 0.54, 95% Cis [.382, 2.527], *p* = .008) and fan type (B = 1.51, SE = 0.35, 95% CIs [.810, 2.214], *p* < .001), as well as their interaction (B = −1.49, SE = 0.66, 95% CIs [−2.811, −.178], *p* = .027), *R*^*2*^ = .35, *F*(5, 80) = 8.54, *p* < .001. A main effect of gender has almost reached the level of statistical significance (B = −0.82, SE = 0.41, 95% CIs [−1.643, .001], *p* = .050), and the effect of age was not significant (*p* = .152) (see SI Table S3 for full results).

Consistent with the pattern for self-reported past violence, the only difference within fan groups was that fused general fans were more likely to endorse future fighting and dying for the group than the non-fused general fans (Figure 1). As the sample was largely male (88%) and there were floor effects for female violence, we re-ran analyses on males only and the significance was unchanged with highly similar main effects of fusion and fan type and significant interaction effects fusion × fan type on past violence and willingness to fight and die, respectively.

## Method: study 2

### Sample

We recruited a total of 203 Australian soccer fans, including general fans of two major Australian teams (*n* = 101) and those who self-identified as ultras (*n* = 101) (Western Sydney Wanderers and Sydney FC; with ultras from the Red and Black Bloc and The Cove, respectively). An online questionnaire was advertised across social media and online platforms, e.g., Facebook, Twitter, Reddit, University participation websites. Additionally, key spokespeople of each active fan group were approached and provided with information about the study to distribute to fellow fans. Participants were incentivized with an AUD$15 voucher.

One participant was excluded from analyses as they indicated at the end of their questionnaire that they had confused the scale points in some measures. Of the final 202 participants (*M*_age_ = 29.91, *SD* = 11.24, range = 18–63), 77% were male (20% female, 3% declined to answer). In terms of education, cultural heritage, and social class, participant demographics approximately reflected trends from the Australian Bureau of Statistics (2016). Adequate sample sizes were determined using a priori power analyses in G*power for anticipated medium effects with a power of .80 at the error probability of .05 (Faul et al., 2009). A minimum of 102 participants was needed for *t*-tests, a minimum of 122 participants was needed for chi-square tests, and a minimum of 165 participants was needed for regression analyses. Ethical approval was obtained from [REDACTED] in accordance with the procedures laid down by the University for ethical approval of all research involving human participants (H10435).

### Measures

First, we asked participants about team affiliation and fan type. Next, we tested identity fusion using the 7-item verbal scale (Gómez et al., 2011) with “[team] members” as the target, including items such as *“[My group] is me”* and “*I am strong because of [my group]”* on a 7-point Likert-type scale (strongly disagree vs. strongly agree), *α* = .87. A verbal scale was preferred as there were no time constraints, however the measure is comparable to the pictorial version used in Study 1 (Gómez et al., 2011). Consequently, fan fusion was analyzed as a continuous variable. Behavioral outcomes were then assessed using binary (yes/no) self-report measures, which included fighting with rival fans, physically abusing rival fans, bans from soccer matches, or prosecutions related to soccer. A total of these items were computed (*α* = .62), but due to a floor effect and very low levels of violence among Australian fans (*M* = 0.17, *SD* = 0.54), we decided to compute a binary outcome measure labeled anti-social behavior (ASB). Reporting “yes” to any of the abovementioned behavioral outcomes was labeled as anti-social behavior (1 = ASB, 0 = no ASB). An adapted version of the four-item centrality subscale of Cameron’s (2004) social identification scale (e.g., “I often think about the fact that I am a member of Sydney FC [Western Sydney Wanderers]”) created an index of ingroup identification ranging, also on a 7-point Likert-type scale (strongly disagree vs. strongly agree) (α = .73). SDO was measured with the short SDO scale (Pratto et al., 2013) (10-point Likert type scale), *α* = .77. Fusion and identification from this dataset are analyzed and contrasted in relation to outgroup anxiety and prejudice in a related paper (REDACTED).

## Results: study 2

In preliminary analyses, we found that younger participants scored higher for both fusion (*r* = −.26, *p* < .001) and SDO (*r* = −.23, *p* < .001), but there was no relationship between age and anti-social behavior (*p* = .904). Men reported higher levels of SDO (*r* = .17, *p* = .013), and past ASD (*r* = .16, *p* = .022) than women. There were no significant correlations between the main variables of interest and education, ethnicity, or class. Fusion correlated significantly with identification and was also significantly related to anti-social behavior and fan type, whereas SDO was only significantly correlated with identification, but no other key variables (see Table S4, SI for full results).

### Associations between fusion and anti-social behaviour

**Figure 2.**
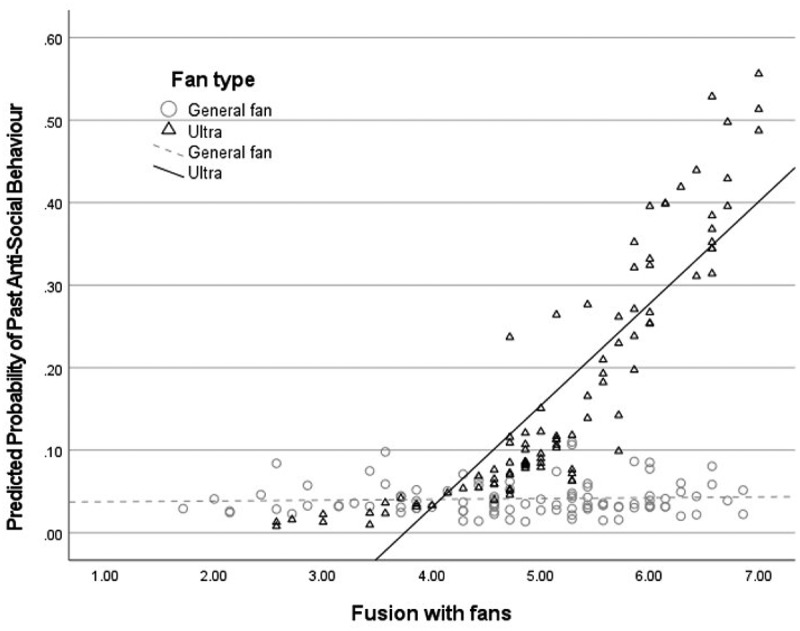
Scatter plot of predicted probability for self-reported past anti-social behaviour based on fan type, fusion, age and gender.

*Note*. Predicted probability of past anti-social behavior based on fan group, fusion, age, and gender

First, we investigated differences between general fans and ultras. Ultras were significantly more fused and likely to report anti-social behavior toward rivals than general fans but scored equally for SDO and identification (Table 2). Next, we used logistic regression analyses to explore how fusion, fan type, age, and gender would predict self-reported past anti-social behavior. We found that ultras (OR = 5.06, 95% CIs [1.497, 17.120], *p* = .009) and more fused fans (OR = 2.13, 95% CIs [1.236, 3.654], *p* = .006) reported more anti-social behavior than general and less fused fans (see Table S5, Model 1, SI for full results). Next, we tested if there would be a significant interaction between fusion and fan type and added the respective interaction term to the model. The fusion × fan type interaction term approached statistical significance (OR = 0.40, 95% CIs [.133, .179], *p* = .091), indicating a trend toward higher levels of fusion being associated with more violence among ultras but not among general fans (Table S5, Model 2, SI for full results; Figure 2).

**Table 2.** Comparing key variables between general and extreme fans in study 2.

	General fans (*M, SD)/*%/*N*	Ultras (*M, SD)/*%/*N*	Test
Fusion	4.76 (1.24)	5.20 (1.03)	*t*(200) = 2.74, *p* = .007, *d* = .39
SDO	2.73 (1.90)	2.97 (2.00)	*t*(197) = −.86, *p* = .393, *d* = .12
Identification	5.34 (0.96)	5.51 (1.02)	*t*(200) = 1.23, *p* = .220, *d* =.17
ASB	6%	20%	χ^2^ (202, 1) = 8.65, *p* = .003, φ_*c*_ = .21

*Note*. ASB = Anti-social Behavior, SDO = Social Dominance Orientation.

For continuous measures, we present means (*M*) and standard deviations (*SD*), for binary measures we present shares (%) of the sample populations *n* coded as 1 out of the total population *N.*

### Testing competing predictors of anti-social behaviour

Finally, we tested if fusion would be a significant predictor of anti-social behavior even after accounting for SDO and identification. After entering all predictors into a logistic regression model simultaneously, we found that fusion predicted anti-social behavior significantly (OR = 2.26, 95% CIs [1.162, 4.393], *p* =.016), as did SDO (OR = 1.08, 95% CIs [1.004, 1.169], *p* = .038), but not identification (OR = 1.07, 95% CIs [.550, 2.075], *p* = .846), *X*^2^(6) = 26.51, Nagelkerke *R*^2^ = .26, *p <* .001 (see Table S5 in SI for full results).

To further examine whether SDO or identification could better explain past anti-social behavior than fusion, we ran two separate regression models replacing fusion for SDO and identification, respectively, including their interactions with fan type. In both models, there were only significant main effects of fan type (*p’s <* .009), but no other main effects or interactions relating to SDO or identification (see Tables S6-S7 in SI for full results).

## Discussion

In two distinct cultural contexts, members of hardcore fan subgroups (ultras) were more likely to have engaged in violence, to indicate willingness to fight for their group in the future (Study 1), and to report past anti-social behavior (Study 2) (H1). We further demonstrated that identity fusion was significantly associated with anti-social behavior (H2a) and there was some evidence that this helped to explain the association between membership of an ultras group and violence (H2b), even when controlling for variables found to be highly relevant for intergroup behaviors in previous studies (H3), i.e., group identification and SDO (Besta et al., 2015).

Our finding that fusion outperformed both SDO and identification as a predictor of anti-social behavior underscores fusion’s value in explaining extreme group behaviors. This adds to the literature on identity fusion’s predictive validity in contrast with identification (Gómez et al., 2011; Swann et al., 2009), as well as in contrast to political ideologies such as SDO, in explaining extreme behaviors and extremism (e.g., Kunst et al., 2019).

### Cultural variations

There were notable similarities and some differences in the Indonesian and Australian findings. In both contexts, fusion and fan type (being an ultra) predicted aggressive behaviors. However, there were differences in the interactive effects of these two variables. First, in Indonesia, fusion significantly influenced violence only among general fans; ultras displayed violent behavior regardless of fusion. However, in Australia, we found a trend toward higher levels of fusion being associated with more violence among ultras, but not among general fans, as has been the case in the previous work in Brazil (Newson et al., 2018). For Indonesian fans, fan type was associated with violence independently from, and in some cases more powerfully than, fusion. It is worth noting that fused general fans were more inclined to engage in violence than non-fused general fans, although the small *n*’s suggest more research with general fans is needed to replicate this finding, and results should be treated cautiously.

A possible explanation for this difference between the two national contexts could be their histories of fan culture. Soccer-related violence is rare in Australia and thus admitting to anti-social behaviors in the context of soccer might be less harmful to the club’s reputation, compared to Indonesia and its long history of soccer-related violence and death (Knijnik & Newson, 2021). Ultras, who were likely to be highly fused to their club, will do what they perceive to be right for the group, which may also involve a curtailing of violence. Again, this speaks to the need to work closely with ultras groups – in this case to understand precisely what it is they feel is “best” for the group and how the use of violence can be discussed as having negative consequences for the wider group.

Another potential reason for the disparity in fusion × subgroup interactions could be the data collection context in Indonesia (i.e., interview-based surveys in a conservative country). The design may have suffered from experimenter effects, due to collecting sensitive data around violence in person. Violence may have been especially under-reported among fused group members who particularly cared about their group’s reputation. However, in relation to the wider literature, inconsistency in the fusion and parochial altruism association has previously been reported in Indonesia, which may relate to wider Indonesian, Southeast Asian, or perhaps majority Muslim country values (Kavanagh et al., 2019). Further research into fusion and membership to violence condoning groups within Indonesia and other Southeast Asian countries is much needed.

### Theoretical and practical implications

Taken together, our findings point toward the role of ultras cultures within the wider, transnational fan community, as they represent the subgroup most prominently associated with extreme group behaviors, in both our studies as well as the wider literature (e.g., Testa & Armstrong, 2010; Heriyanto, 2018). Our focus here was on multiple anti-social behaviors, and, in line with previous findings, the potential for engagement in violent behaviors (both past and future) was significantly higher among ultras compared to general fans not involved in ultras culture.

Nonetheless, the association between subgroups and disruptive or violent behaviors is not necessarily true for all ultras (Lavalette & Mooney, 2013). Indeed, research has shown that subgroup membership of ultras overlaps with both negative and positive extreme behaviors (e.g., Jack, 2021; Knijnik, 2020) and that variation between fan movements across cultures is likely (Spaaij, 2007). We strongly advocate for international security and policing, such as at World Cup events, to avoid homogenizing fan groups. By valuing localized soccer cultures in global settings, and ideally including elements of co-design with the diverse fan groups represented, international events have the best chances of being peaceful.

Speaking to such cross-cultural differences, our findings reveal how identity fusion may be an underlying group-based mechanism that can help us better understand why some fans are more likely to engage in extreme behaviors than others. As such, the findings offer a theoretical contribution to the elaborated social identity model (ESIM) (Stott et al., 2020) and goes further to elucidate the critical role of identity fusion in predicting extreme group behaviors, seemingly surpassing traditional constructs such as social identification and social dominance orientation (SDO) (Cialdini et al., 1991; Gómez et al., 2011; Swann et al., 2009). By examining hardcore fan subgroups (ultras) across distinct cultural contexts – Australia and Indonesia – the study reveals that identity fusion is a more potent predictor of anti-social behavior than traditional metrics, though its impact varies with cultural and historical contexts (Heriyanto, 2018; Knijnik & Newson, 2021). This finding challenges the universality of previous models and underscores the necessity for culturally nuanced approaches in understanding and managing group-related violence, suggesting that identity fusion’s role in extreme behaviors warrants further exploration.

We sought to further strengthen the existing literature on the relation between identity fusion and extreme pro-group action by using an approach that relied on less Western, Educated, Industrialised, Rich, and Democratic (WEIRD; Henrich et al., 2010) participants and more on Worldwide, In Situ, Local, and Diverse (WILD; Newson et al., 2020) participants. We achieved this by including real-world soccer fans (including hard to access ultras), working with collaborators with special interest and local knowledge of these populations and working in contrasting cultures that go beyond typical samples of Northern European and North American participants.

Intergroup violence is unlikely to be motivated by identity fusion alone – quite the opposite, in fact: many violent Indonesian fans, largely members of extreme fan groups, reported low levels of fusion. Here, it may have been the violence-condoning norms of longstanding extreme or ultras supporter groups that related to elevated rates of violence, rather than fusion with club (Cialdini et al., 1991; Louis, 2014). Whatever the context, tapping into the *pro-social* motivations of highly fused fans could help to promote more peaceful norms among high-risk fan groups, perhaps by emphasizing successful fan groups who are known for their peaceful conduct and impressive fan displays or promoting children and young people’s access to fan culture and their need for more peaceful environments. For example, Germany’s “Nationales Konzept Sport und Sicherheit” (National Sport Concept and Safety) and related fan-projects in and around stadiums have proven successful in reducing violence (NKSS, 2012). Furthermore, qualitative findings from Turkey suggest that the most fused fans focus on wider social issues and prevent violence in some instances (Reyhan, 2019).

Club management and police forces could encourage more socially desirable behaviors by identifying and working closely with the most fused fans to (a) better understand high-risk groups and (b) encourage fused fans to lead by example, instilling pro-social behaviors with club-level support. Such endeavors are multi-generational, requiring long-term planning and commitment from clubs and police forces. As such, dedicated soccer officers and funded supporter liaison officers (SLOs, such as those now found at all large European clubs as legislated by UEFA) are invaluable when establishing a network that traverses fan groups, club management, and security/policing.

Homogenizing ultras by labeling and penalizing them *en masse*, when only a minority are violent, will only stigmatize these groups and push them further away from mainstream soccer culture (Lavalette & Mooney, 2013; Stott & Pearson, 2007). Instead, harnessing the group commitment found among fused supporters could reduce willingness to engage in violence, insofar as those who most passionately care about the fate of their clubs may also be more concerned about protecting their group’s reputation by avoiding penalties for acts of hooliganism.

In addition, normative differences may help to account for the roles of gender and age in expressed desires for outgroup anti-social behavior, given that aggressiveness is typically more strongly reinforced in young males by cultural stereotypes and role models (Eagly & Wood, 1991; Nivette et al., 2019). Actively encouraging and supporting access to games for women, children, and the elderly via ticket sales and safe stadia could therefore help to bring about a change in cultural values among higher-risk groups, as well as being financially lucrative. One potential risk here is that the “protect and defend” mentality among highly fused fans, who perceive other fans as kin (Newson et al., 2018), could be further triggered with more women and children present, leading to more intergroup anti-social behavior. All these additional factors warrant further empirical investigation to disambiguate the relative power of norms and strong forms of group alignment in predicting patterns of outgroup violence.

## Conclusion

Only a minority of soccer fans engage in violence. Most soccer fans surveyed reported histories unmarred by anti-social behavior. Ultras, however, were typically more violent than general fans in both Indonesia and Australia, making this identity the biggest predictor of anti-social behavior. We found that identity fusion was also associated with fan violence. However, its effects differed across cultures: when combined with subgroup membership, there were trends for fusion to predict anti-social behaviors among general fans in Indonesia and among ultras in Australia. Compared to subgroup membership and fusion, social identification and social dominance orientation did little to explain anti-social behaviors at an individual level. This research could be used to help supporter liaison officers, dedicated police officers, and other specialists trained to work with extreme soccer fans, to harness group identities for more peaceful outcomes, such as more self-policing within groups that have a history of spreading violence-condoning norms (Stott et al., 2019), thus further strengthening the valuable offerings from the ESIM.

Research on soccer fans can help shed light on the causes of societally negative behaviors associated with both subgroup membership, and extreme social identities associated with the violence found among a much wider range of gangs, politically ideological groups, and religious or ethnic conflicts. Since factors such as subgroup membership and identity fusion play a key role in many such contexts, research on this topic can help us to better understand and address the factors that lead to anti-social behavior and violence.

## Supplementary Material

Si.docx

## Data Availability

The data that support the findings of this study are openly available on the Open Science Framework at https://osf.io/j3zg8/?view_only=e6af5ea88b07466fa2b530f874972110.
