# Supplementary material for: Anti-social behavior and soccer identities: different continents, same mindset?
Source: Self Identity. 2024 Nov 12;23(7-8):616–33. doi: 10.1080/15298868.2024.2423829 (PMC11627210; doi:10.1080/15298868.2024.2423829)
Supplement: Si.docx [file PSAI_A_2423829_SM5072.docx]

**Supplementary Information**

**Table S1**

*Descriptive Statistics and Correlations of Key Variables in Study 1*

|  | *M (SD)* | %/N |  | 1 | 2 | 3 |
| --- | --- | --- | --- | --- | --- | --- |
| 1 Fusion | - | 35% |  |  |  |  |
| 2 Fight and die | 4.59 (1.45) | - |  | .36^***^ |  |  |
| 3 Past violence | - | 22% |  | .20 | .40^***^ |  |
| 4 Fan type | - | 48% |  | .47^***^ | .48^***^ | .33^**^ |

*Note*. Fusion (fused = 1, not fused = 0); Past violence (yes = 1, no = 0); Fan type (ultra = 1, general = 0). Skew and kurtosis ranged -1.98 – 1.88 (SEs .17 - .48).

For continuous measures we present means (*M*) and standard deviations (*SD*), for binary measures we present shares (%) of the sample populations *n* coded as 1 out of the total population *N*.
^***^ *p* < .001, ^**^ *p*< .01, ^*^ *p* < .05.

**Table S2**

*Logistic Regression Models Predicting Self-Reported Violent Behaviour in Study 1*

| Variable | *B* | *SE* | *Wald* | *p* | *Exp(B)* | *95%CIs* |
| --- | --- | --- | --- | --- | --- | --- |
| (Intercept) | 20.10 | 1151.75 | <0.01 | .999 |  |  |
| Age | 0.12 | 0.05 | 5.19 | .023 | 1.12 | [1.016, 1.240] |
| Gender | -19.67 | 11516.75 | <0.01 | .999 | .00 |  |
| Fusion | 2.72 | 1.24 | 4.77 | .029 | 15.16 | [1.323, 173,770] |
| Fan type | 2.80 | 0.97 | 8.33 | .004 | 16.36 | [2.451, 109.232] |
| Fusion * fan type | -3.24 | 1.45 | 4.96 | .026 | .04 | [.002, .678] |
|  | *X*^2^(7) = 23.77, Nagelkerke *R*^2^ = .37, *p <* .001 | | | | | |

*Note*. Gender = male (0), female (1). Fan type = general fan (0), ultra (1).

**Table S3**

*Linear Regression Models Predicting Future Willingness to Fight and Die in Study 1*

| Variable | *B* | *SE* | *β* | *t* | *p* | *95%CIs* |
| --- | --- | --- | --- | --- | --- | --- |
| (Intercept) | 3.88 | 0.20 |  | 19.03 | < .001 | [3.472, 4.283] |
| Age | 0.03 | 0.02 | .13 | 1.45 | .152 | [-.013, .079] |
| Gender | -0.82 | 0.41 | -.18 | -1.99 | .050 | [-1.643, .001] |
| Fusion | 1.46 | 0.54 | .48 | 2.70 | .008 | [.382, 2.527] |
| Fan type | 1.51 | 0.35 | .52 | 4.29 | < .001 | [.810, 2.214] |
| Fusion * fan type | -1.49 | 0.66 | -.47 | -2.26 | .027 | [-2.811, -.178] |
|  | *R^2^* = .35, *F*(5, 80) = 8.54, *p* < .001 | | | | | |

*Note*. Gender = male (0), female (1). Fan type = general fan (0), ultra (1).

**Table S4**

*Correlations and Descriptive Statistics of Key Variables in Study 2*

|  | M (SD) | %/*N* |  | 1 | 2 | 3 | 4 | 5 |
| --- | --- | --- | --- | --- | --- | --- | --- | --- |
| 1. Fusion | 4.98 (1.16) |  |  | - |  |  |  |  |
| 2. SDO | 2.85 (1.94) |  |  | .01 | - |  |  |  |
| 3. Identification | 5.42 (0.99) |  |  | .58^***^ | -.29^***^ | - |  |  |
| 4. ASB |  | 13% |  | .16^*^ | .12 | .08 | - |  |
| 5. Fan type |  | 50% |  | .19^**^ | .06 | -.09 | .21^**^ | - |

*Note.* SDO = Social Dominance Orientation, ASB = Anti-Social Behaviour. Fan type = general fan (0), ultra (1). Variables consist of the following ranges: fusion (1-7); ASB; Skew and kurtosis ranged -0.8 – 1.2 (SE .17 - .40). For continuous measures we present means (*M*) and standard deviations (*SD*), for binary measures we present shares (%) of the sample populations *n* coded as 1 out of the total population *N* ^***^ *p* < .001, ^**^ *p* < .01, ^*^ *p* < .05.

**Table S5**

*Logistic Regression Models Predicting Self-Reported Anti-Social Behaviour in Study 2*

|  | *Model 1* | | | | | | *Model 2* | | | | | | *Model 3* | | | | | |
| --- | --- | --- | --- | --- | --- | --- | --- | --- | --- | --- | --- | --- | --- | --- | --- | --- | --- | --- |
| Variable | *B* | *SE* | *Wald* | *p* | *Exp(B)* | *95%CIs* | *B* | *SE* | *Wald* | *p* | *Exp(B)* | *95%CIs* | *B* | *SE* | *Wald* | *p* | *Exp(B)* | *95%CIs* |
| (Intercept) | -1.70 | 0.35 | 24.25 | <.001 |  |  | -1.93 | 0.41 | 21.85 | <.001 |  |  | -3.67 | 0.67 | 30.37 | <.001 |  |  |
| Age | 0.04 | 0.02 | 2.62 | .105 | 1.04 | [.992, 1.092] | 0.03 | 0.03 | 1.89 | .169 | 1.04 | [.985, 1.087] | 0.06 | 0.03 | 4.52 | .033 | 1.06 | [1.005, 1.118] |
| Gender | -0.75 | 0.80 | 0.88 | .349 | 0.48 | [.100, 2.259] | -0.69 | 0.80 | 0.73 | .392 | .502 | [.104, 2.429] | -0.47 | 0.82 | 0.32 | .571 | .63 | [.126, 3.136] |
| Fusion | 0.75 | 0.28 | 7.44 | .006 | 2.13 | [1.236, 3.654] | 1.06 | 0.36 | 8.85 | .003 | 2.90 | [1.437, 5.832] | 0.82 | 0.34 | 5.77 | .016 | 2.26 | [1.162, 4.393] |
| Fan type | 1.62 | 0.62 | 6.80 | .009 | 5.06 | [1.497, 17.120] | 1.24 | 0.69 | 3.26 | .071 | 3.46 | [.899, 13.296] | 1.83 | 0.67 | 7.50 | .006 | 6.21 | [1.680, 22.929] |
| Fusion * fan type | - | - | - | - | - | - | -0.93 | 0.56 |  | .091 | .40 | [.133, .179] | - | - | - | - | - | - |
| Identification | - | - | - | - | - | - | - | - | - | - | - | - | 0.07 | 0.34 | 0.04 | .846 | 1.07 | [.550, 2.075] |
| SDO | - | - | - | - | - | - | - | - | - | - | - | - | 0.08 | 0.04 | 4.29 | .038 | 1.08 | [1.004, 1.169] |
|  | *X*^2^(4) = 21.75, Nagelkerke *R*^2^ = .21, *p <* .001 | | | | | | *X*^2^(5) = 24.38, Nagelkerke *R*^2^ = .24, *p <* .001 | | | | | | *X*^2^(6) = 26.51, Nagelkerke *R*^2^ = .26, *p <* .001 | | | | | |

*Note*. Gender = male (0), female (1). Fan type = general (0), ultra (1).

**Table S6**

*Logistic Regression Models Predicting Self-Reported Anti-Social Behaviour Based Social Dominance Orientation Instead of Identity Fusion in Study 2*

|  | *Model 1* | | | | | | *Model 2* | | | | | |
| --- | --- | --- | --- | --- | --- | --- | --- | --- | --- | --- | --- | --- |
| Variable | *B* | *SE* | *Wald* | *p* | *Exp(B)* | *95%CIs* | *B* | *SE* | *Wald* | *p* | *Exp(B)* | *95%CIs* |
| (Intercept) | -3.32 | 0.59 | 31.43 | <.001 | .04 |  | -3.40 | 0.63 | 28.93 | <.001 | .03 |  |
| Age | 0.04 | 0.02 | 3.15 | .076 | 1.04 | [.995, 1.095] | 0.04 | 0.02 | 3.14 | .077 | 1.04 | [.995, 1.095] |
| Gender | -0.62 | 0.80 | 0.59 | .442 | .54 | [.113, 2.594] | -0.62 | 0.80 | 0.59 | .442 | .54 | [.113, 2.589] |
| Fan type | 1.88 | 0.65 | 8.45 | .004 | 6.56 | [1.845, 23.320] | 1.98 | 0.69 | 8.26 | .004 | 7.22 | [1.876, 27.815] |
| SDO | 0.05 | 0.03 | 2.67 | .102 | 1.05 | [.990, 1.122] | 0.08 | 0.06 | 1.70 | .192 | 1.09 | [.959, 1.230] |
| SDO * fan type |  |  |  |  |  |  | -0.04 | 0.07 | 0.31 | .579 | .96 | [.836, 1.105] |
|  | *X*^2^(4) = 15.45, Nagelkerke *R*^2^ = .15, *p* = .004 | | | | | | *X*^2^(5) = 15.75, Nagelkerke *R*^2^ = .16, *p* = .008 | | | | | |

*Note*. Gender = male (0), female (1). Fan type = general (0), ultra (1).

**Table S7**

*Logistic Regression Models Predicting Self-Reported Anti-Social Behaviour Based on Identification Instead of Identity Fusion in Study 2*

|  | *Model 1* | | | | | | *Model 2* | | | | | |
| --- | --- | --- | --- | --- | --- | --- | --- | --- | --- | --- | --- | --- |
| Variable | *B* | *SE* | *Wald* | *p* | *Exp(B)* | *95%CIs* | *B* | *SE* | *Wald* | *p* | *Exp(B)* | *95%CIs* |
| (Intercept) | -3.14 | 0.55 | 32.35 | <.001 | .04 |  | -3.12 | 0.56 | 31.55 | <.001 | .04 |  |
| Age | 0.03 | 0.02 | 1.31 | .252 | 1.03 | [.980, 1.078] | 0.03 | 0.02 | 1.27 | .260 | 1.03 | [.980, 1.078] |
| Gender | -0.89 | 0.79 | 1.29 | .257 | .41 | [.088, 1.914] | -0.89 | 0.79 | 1.28 | .257 | .41 | [.088, 1.917] |
| Fan type | 1.69 | 0.62 | 7.37 | .007 | 5.42 | [1.599, 18.377] | 1.66 | 0.64 | 6.81 | .009 | 5.28 | [1.513, 18.392] |
| Identification | 0.35 | 0.27 | 1.68 | .195 | 1.41 | [.838, 2.388] | 0.24 | 0.59 | 0.17 | .683 | 1.28 | [.398, 4.085] |
| Identification * fan type |  |  |  |  |  |  | 0.13 | 0.67 | 0.04 | .846 | 1.14 | [.309, 4.194] |
|  | *X*^2^(4) = 14.59, Nagelkerke *R*^2^ = .15, *p = .006* | | | | | | *X*^2^(5) = 14.63, Nagelkerke *R*^2^ = .15, *p =* .012 | | | | | |

*Note*. Gender = male (0), female (1). Fan type = general (0), ultra (1)
